# Supplementary material for: Seeing Things: A Community Science Investigation into Motion Illusion Susceptibility in Domestic Cats (Felis silvestris catus) and Dogs (Canis lupus familiaris)
Source: Animals (Basel). 2022 Dec 16;12(24):3562. doi: 10.3390/ani12243562 (PMC9774501; doi:10.3390/ani12243562)
Supplement: Supplementary file 1 [file animals-12-03562-s001.zip › animals-2036829-supplementary file.pdf]

Figure S1: Rotating Snakes

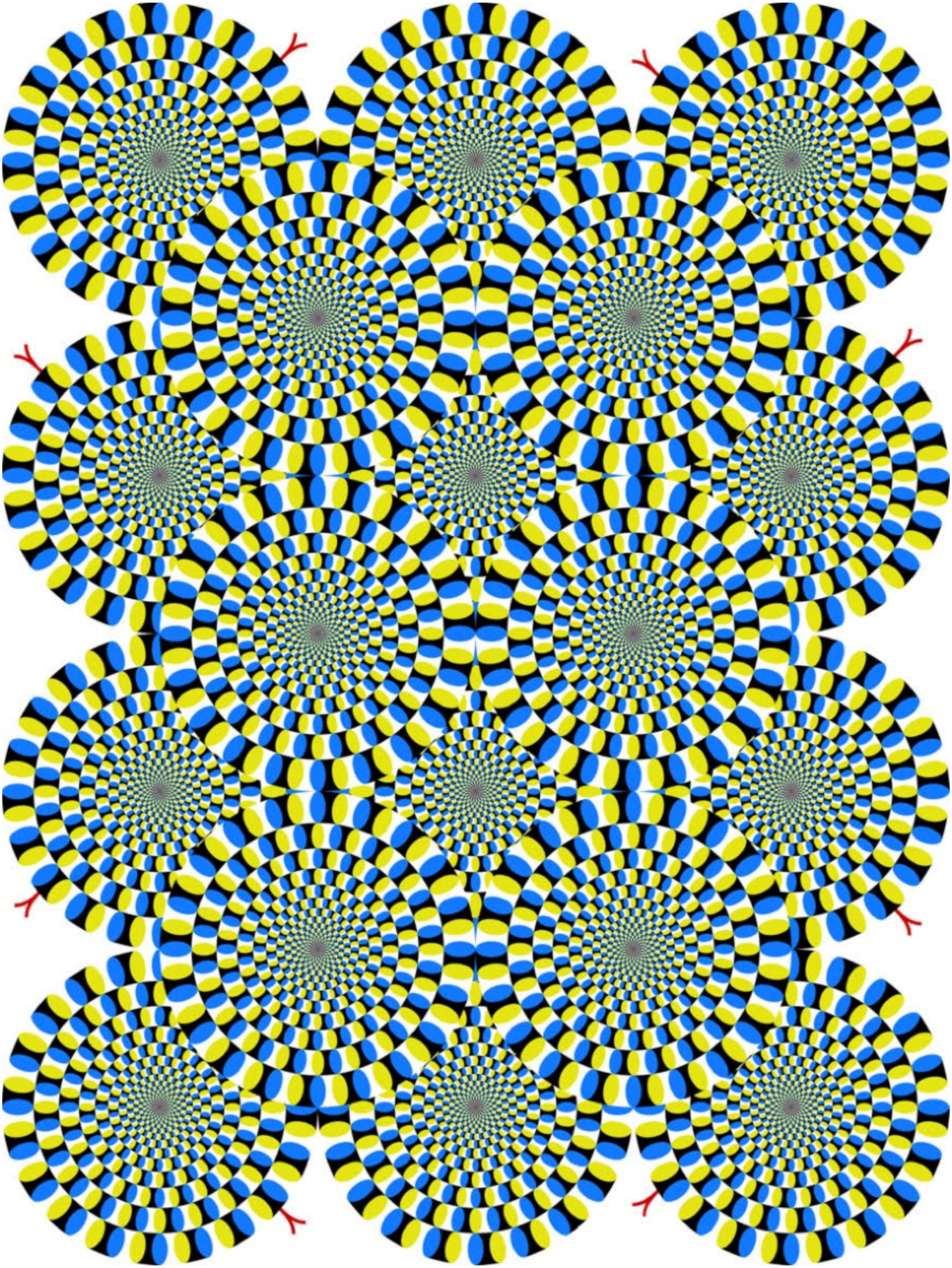

Figure S2: Plain Snakes

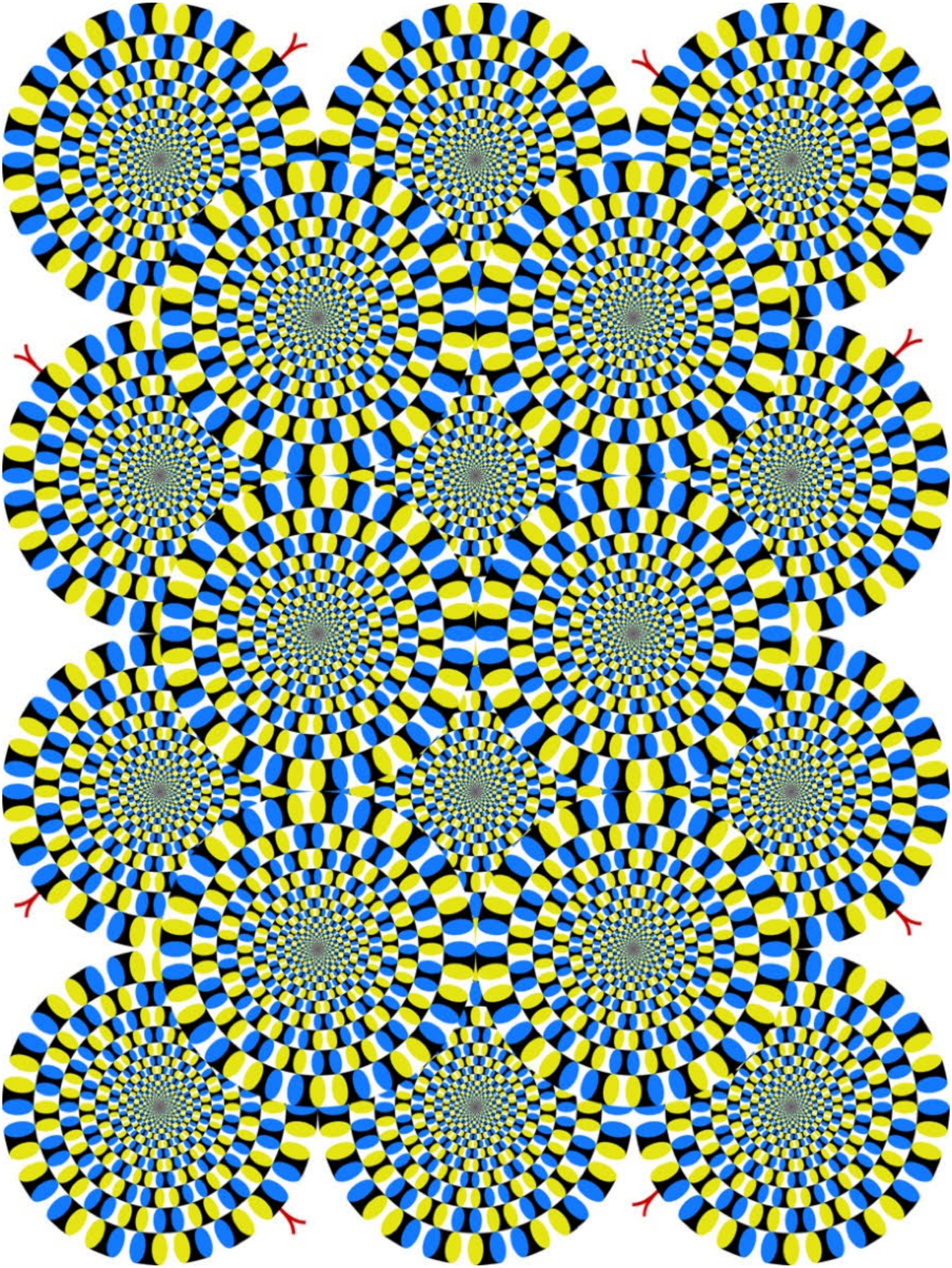

Figure S3: Big Circles

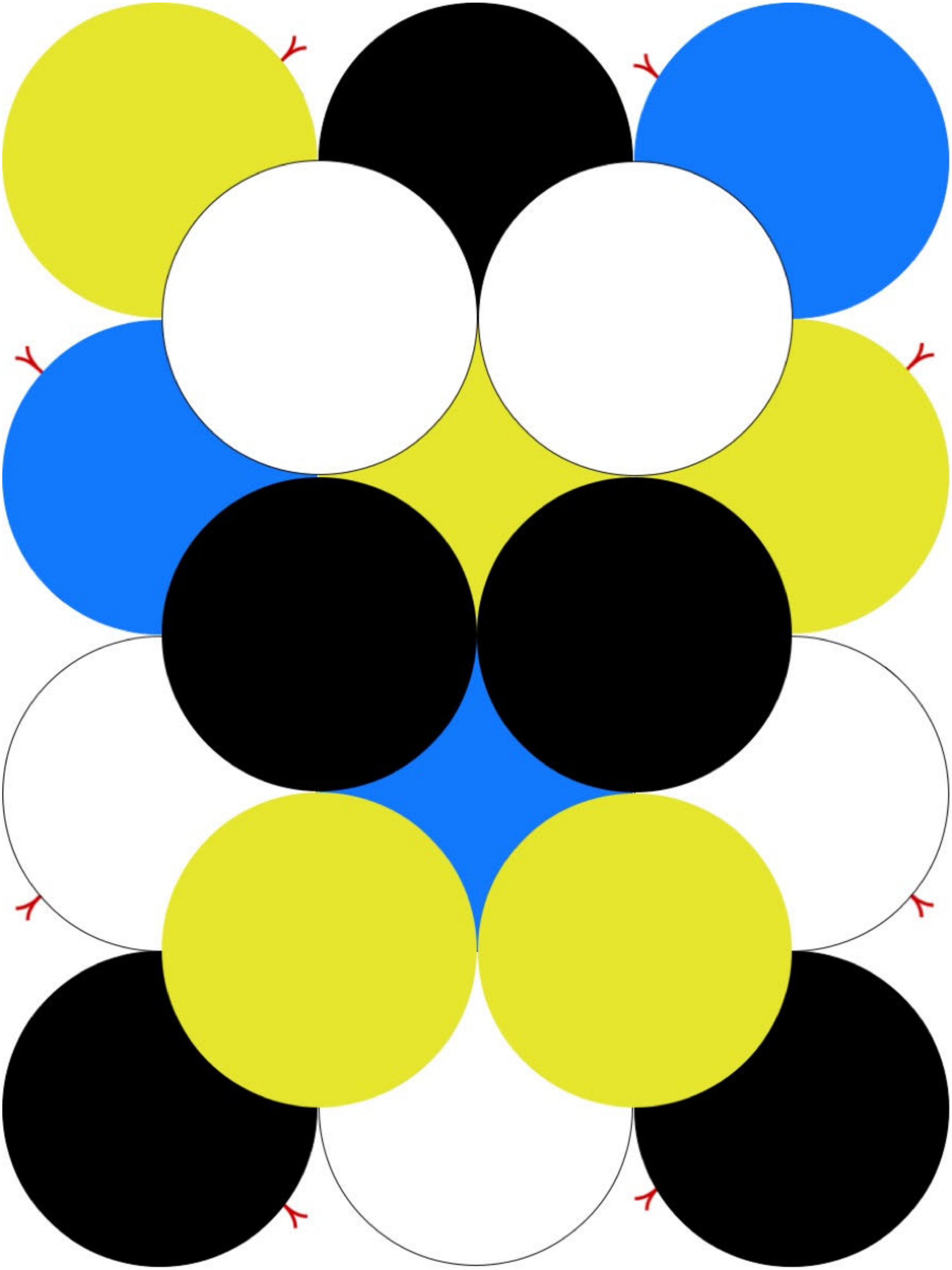

Figure S4: Rotating Snakes

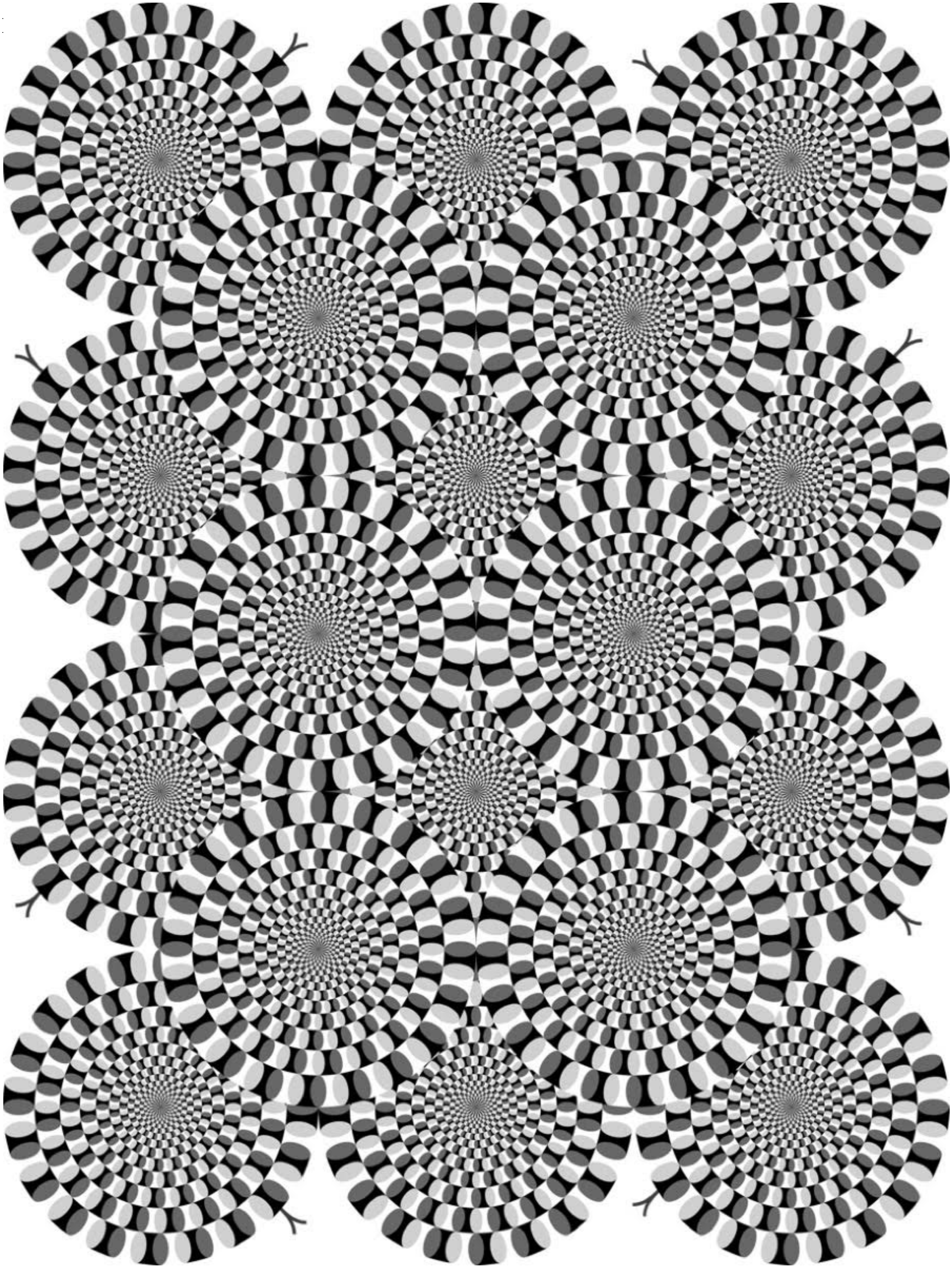

Figure S5: Plain Snakes

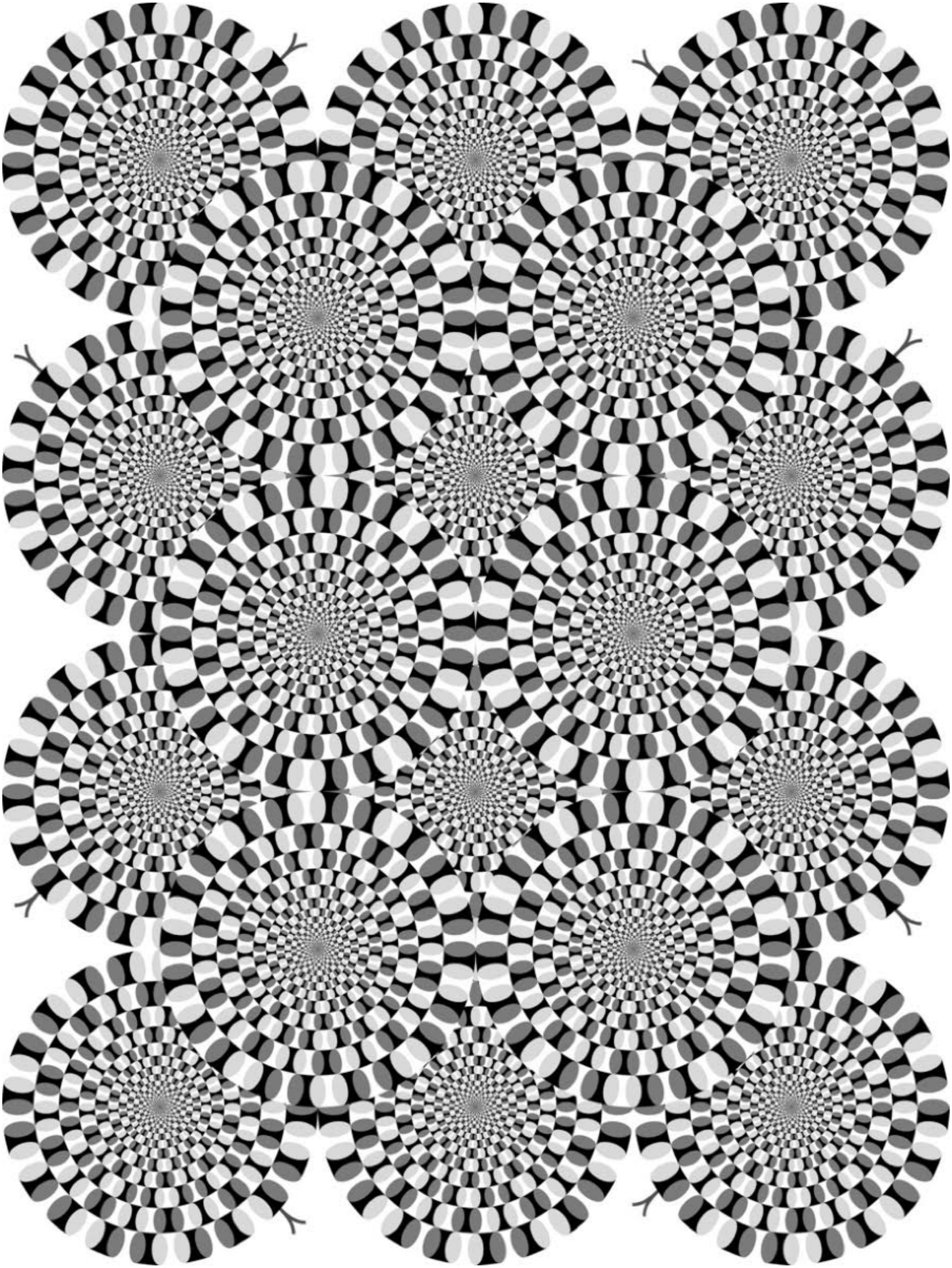

Figure S6: Big Circles

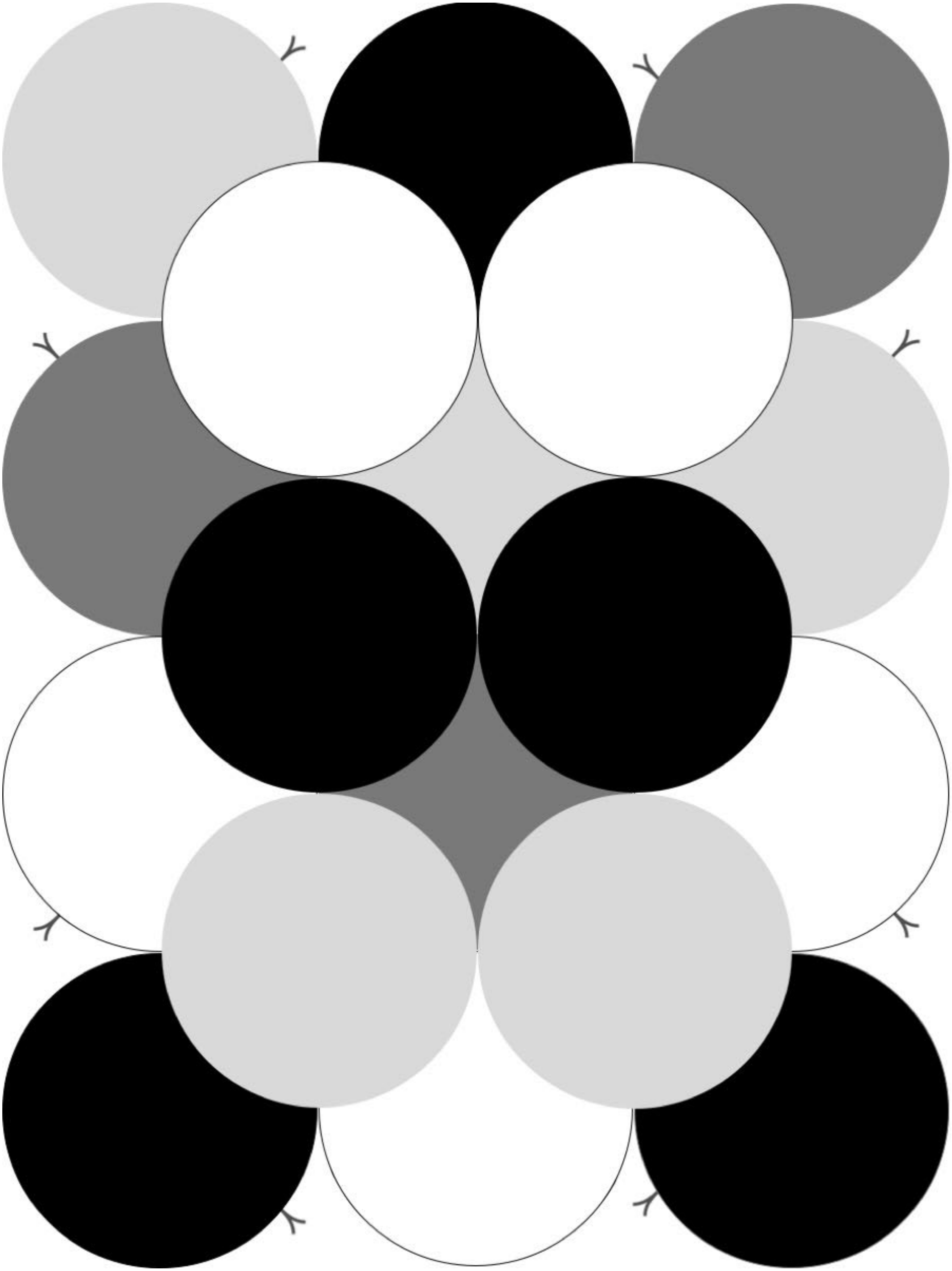

Table S1

Pet Name	Species	Color Ink	Most Time Spent: Rotating Snakes, Plain Snakes, or Big Circles?
Ash	Cat	Color	Rotating Snakes
Baker	Dog	Color	Plain Snakes
Baloo	Cat	Color	Rotating Snakes
Bee	Dog	Color	Rotating Snakes
Berenice	Cat	Color	Rotating Snakes
Bongo	Dog	B&W	Plain Snakes
Bubba	Cat	B&W	Rotating Snakes
Buddy	Dog	Color	N/A
BunBun	Cat	Color	Rotating Snakes
Buzz	Cat	Color	N/A
Cassie	Dog	B&W	Rotating Snakes
Charlie	Dog	Color	Rotating Snakes
Chloe	Dog	B&W	Rotating Snakes
Chouqui	Cat	Color	Big Circles
Cookie	Dog	B&W	Big Circles
Cornelius	Cat	Color	Big Circles
Cutacha	Dog	Color	Rotating Snakes
Daisy Mae	Dog	Color	N/A
Daphne	Dog	Color	Big Circles
Dobby	Dog	Color	Big Circles
Donut	Cat	B&W	Plain Snakes
Drizzle	Cat	Color	Big Circles
Dulce	Dog	Color	Plain Snakes

Ella	Cat	Color	Plain Snakes
Emma	Cat	Color	Plain Snakes
Fig	Dog	Color	Big Circles
Frederico	Cat	Color	Big Circles
Georgie	Cat	Color	Big Circles
Hamish Herzog Miau-Miau	Cat	B&W	Big Circles
Harriet	Dog	B&W	Big Circles
Hattie	Dog	B&W	Plain Snakes
Hemma	Dog	B&W	Big Circles
Igor	Cat	B&W	Plain Snakes
Jade	Cat	Color	Plain Snakes
Jazz	Dog	Color	Plain Snakes
Jessie	Dog	Color	Plain Snakes
Juhan	Cat	Color	Big Circles
Kebab	Cat	Color	Plain Snakes
Kevin	Dog	Color	Big Circles
Kozak	Dog	Color	Plain Snakes
Kylo	Cat	Color	Plain Snakes
LokiCat	Cat	B&W	Rotating Snakes
LokiDog	Dog	Color	N/A
Maddy	Dog	B&W	Plain Snakes
Marty	Dog	Color	Rotating Snakes
Maui	Cat	Color	Plain Snakes
Micki	Dog	B&W	Plain Snakes
Natasha	Dog	Color	Plain Snakes

Nigel Phillip	Dog	B&W	Big Circles
Orange	Cat	Color	Plain Snakes
Phoebe	Dog	B&W	Plain Snakes
Pogacs	Dog	B&W	Rotating Snakes
Pokey	Cat	Color	Rotating Snakes
Rain	Dog	Color	Big Circles
Robbie	Cat	Color	Plain Snakes
Rosey	Cat	B&W	Plain Snakes
Rosie	Cat	Color	Plain Snakes
Ruby	Cat	B&W	Big Circles
Ruckus	Cat	B&W	Big Circles
Ruido	Dog	Color	Rotating Snakes
Rupert	Cat	B&W	Rotating Snakes
Scout	Dog	B&W	N/A
Shadow	Cat	Color	Plain Snakes
Siggy	Cat	B&W	Big Circles
Skeletor	Cat	B&W	Big Circles
Skittles	Cat	Color	Big Circles
Stella	Cat	B&W	Big Circles
Teddy	Dog	Color	Plain Snakes
TiaCat	Cat	Color	Rotating Snakes
TiaDog	Dog	B&W	N/A
Tonks	Cat	Color	Rotating Snakes
Wally	Cat	B&W	Rotating Snakes
Wendell	Dog	Color	Plain Snakes
Willoughby	Cat	Color	Rotating Snakes

Zedd	Dog	Color	Plain Snakes

[1] N/A denotes no time was spent with stimuli. [2] B&W denotes black and white.
